# Inhibition of the NLRP3-inflammasome as a potential approach for neuroprotection after stroke

**DOI:** 10.1038/s41598-018-24350-x

**Published:** 2018-04-13

**Authors:** Saifudeen Ismael, Liang Zhao, Sanaz Nasoohi, Tauheed Ishrat

**Affiliations:** 1 Department of Anatomy and Neurobiology, The University of Tennessee Health Science Center, Memphis, TN USA; 2 Neuroscience Research Center, Shahid Beheshti University of Medical Sciences, Tehran, Iran; 3 Neuroscience Institute, The University of Tennessee Health Science Center, Memphis, TN USA

## Abstract

Activation of the NOD-like receptor protein (NLRP3)-inflammasome has been postulated to mediate inflammatory responses to brain damage during ischemic/reperfusion (I/R) injury. We therefore hypothesized that MCC950, a selective NLRP3-inflammasome inhibitor provides protection in mouse model of transient middle cerebral artery occlusion (tMCAO). Focal cerebral ischemia was induced by 60 min tMCAO followed by intraperitoneal administration of MCC950 (50 mg/kg) or saline at 1 h and 3 h post-occlusion. After 24 h of I/R, mice were tested for neurological outcome and were sacrificed for the analysis of infarct size and estimating NLRP3-inflammasome and apoptotic markers as well. Spectrophotometric method was used to determine hemoglobin (Hb) content as a marker of intracerebral hemorrhage. MCC950-treated mice showed a substantial reduction in infarction, edema and Hb content compared to saline controls in parallel with improved neurological deficits. MCC950 reduced expression of NLRP3-inflammasome cleavage products Caspase-1 and interlukin-1β (IL-1β) in penumbral region. These protective effects of MCC950 were associated with decreased TNF-α levels as well as poly (ADP-ribose) polymerase (PARP) and Caspase-3 cleavage and paralleled less phosphrylated NFκBp65 and IκBα levels. Taken together, these data indicate that inhibition of NLRP3-inflammasome with MCC950 has therapeutic potential in ischemic stroke models. Further investigations into the therapeutic efficacy and protocols are needed to confirm whether MCC950 treatment could be a promising candidate for clinical trials.

## Introduction

Little has been admitted to medical practice in ischemic stroke, standing as the fifth-leading cause of death and long-term disability in the United States^1^. According to the last updates in accredited database, few medications are available for acute stroke management in conjunction to vascular recanalization and supportive care measures^2,3^. Anti-inflammatory agents have been long in high interest to explore promising approaches for the flamed ischemic tissue^4^ or reperfusion injury consequent to therapeutic revascularization^5,6^. Corticosteroids as unique pluripotent immune-suppressive agents might be of high value in stroke patients^7,8^. Nevertheless, the prevalence of infectious diseases i.e. pneumonia in stroke patients is a concern in chronic administration of the drug^9,10^. As such, exploring new therapies targeting specific but major pro-inflammatory signals in stroke might provide efficiently reliable medical protocols.

Recent findings postulate that signaling of the NOD-like receptor protein (NLRP3) is an essential mechanism in mediating inflammatory responses in aseptic tissue injury during ischemic stroke^11,12^. Sensing several stroke-induced stimuli, the cytosolic pattern recognition receptor NLRP3 recruits the adapter protein the apoptosis-associated speck-like (ASC) pro-caspase-1 leading to caspase-1 production and subsequent interlukin-1 β (IL-1β) maturation and release^13,14^. The significance of pro-inflammatory and pro-apoptotic effects of IL-1 β is quite well-founded in acute stroke^15,16^. Furthermore independent of IL-1 β, the induced caspase-1 leads to pyroptotic cell death which is well established in glial cells to induce massive cytokine release through intramembranous pores^17^. Consistently, several studies indicate that NLRP3 repression improves ischemic insult and neurovascular complications in cellular^18^ and animal models of stroke^19,20^. Nonetheless, mostly dealing with genetic modulation or non-specific neuroprotectants they fail to reflect the clinical advantages. Therefore, this has encouraged efforts to develop novel NLRP3 inhibitors with acceptable biocompatibility for clinical trials. Our recent findings^21^ imply NLRP3 suppression through genetic modulations confers remarkable protection against animal model of stroke. In wake of translation, we aimed to evaluate the therapeutic advantages of the small molecule MCC950. The novel compound MCC950 introduced as a specific anti-inflammatory compound^22^ has been shown to confer protection in CNS disease models e.g. Alzheimer’s disease^23^ or systemic disorders dealing pathological inflammation^24,25^. A recent report has already addressed the protective effect of MCC950 in subacute phase in a photothrombotic stroke^20^. Coupled with its optimal pharmacokinetic characteristics^26^, this may posit MCC950 as a promising candidate for clinical trials in stroke patients. In accordance with Stroke Treatment Academic Industry Roundtable (STAIR) suggestion for rigorous preclinical research and to consider large vessels occlusion models^27,28^, our experimental findings show specific NLRP3 inhibition with MCC950 protect the brain against MCAO in mice.

## Results

### MCC950 treatment attenuates cerebral infraction, edema, hemorrhagic transformation and functional deficit following MCAO

**Figure 1:**
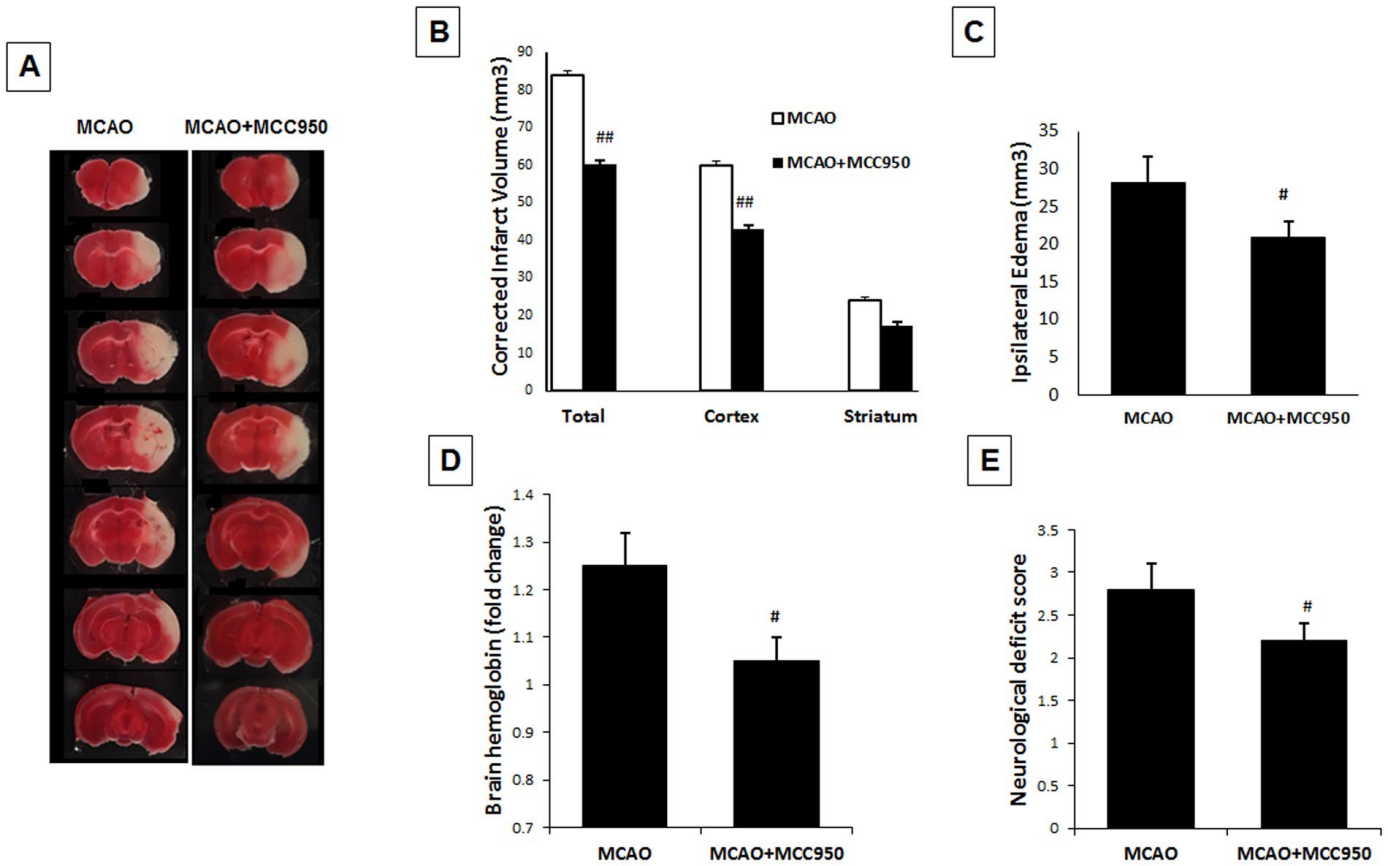
MCC950 treatment reduces infarct size, cerebral edema and hemorrhage as well as functional outcome at 24 h post-MCAO. The representative TTC sections (**A**), MCC950 treatment led to significant reduction in infarct volume (**B**), decrease in ipsilateral edema (**C**) and prevention of intracerebral haemorrhage (**D**). This was in parallel with improved neurological scores (**E**). Values are expressed as mean ± SEM (n = 7–8), ^#^p < 0.05, ^##^p < 0.01 vs saline treated MCAO animals.

As represented in Fig. 1A for TTC sections, mice treated with MCC950 showed significantly (p < 0.01) smaller infarct size (63.9 ± 5.4 mm^3^) compared to saline-treated (82.6 ± 6.4 mm^3^) MCAO animals (Fig. 1B). Such a difference was more prominent in cortical regions overlapping the penumbral area. This was associated with a moderate decrease in the hemispheric swelling (Fig. 1C) in MCAO animals (p = 0.043). To examine the effects of MCC950 on hemorrhage, brain tissue Hb content was estimated as an index for incidence of intracerebral hemorrhage in perfused brains at 24 h after MCAO (Fig. 1D). The Hb content was significantly (P < 0.05) reduced in MCAO + MCC950 group compared to saline-MCAO. To examine the effect of MCC950 on acute functional outcomes, we used neurological deficit scores at 24 h after MCAO (Fig. 1E). The model animals exhibited prominent neurological deficits which were significantly reduced (P < 0.05) with MCC950 treatment.

### MCC950 treatment prevents stroke-induced NLRP3 inflammasome activation

**Figure 2:**
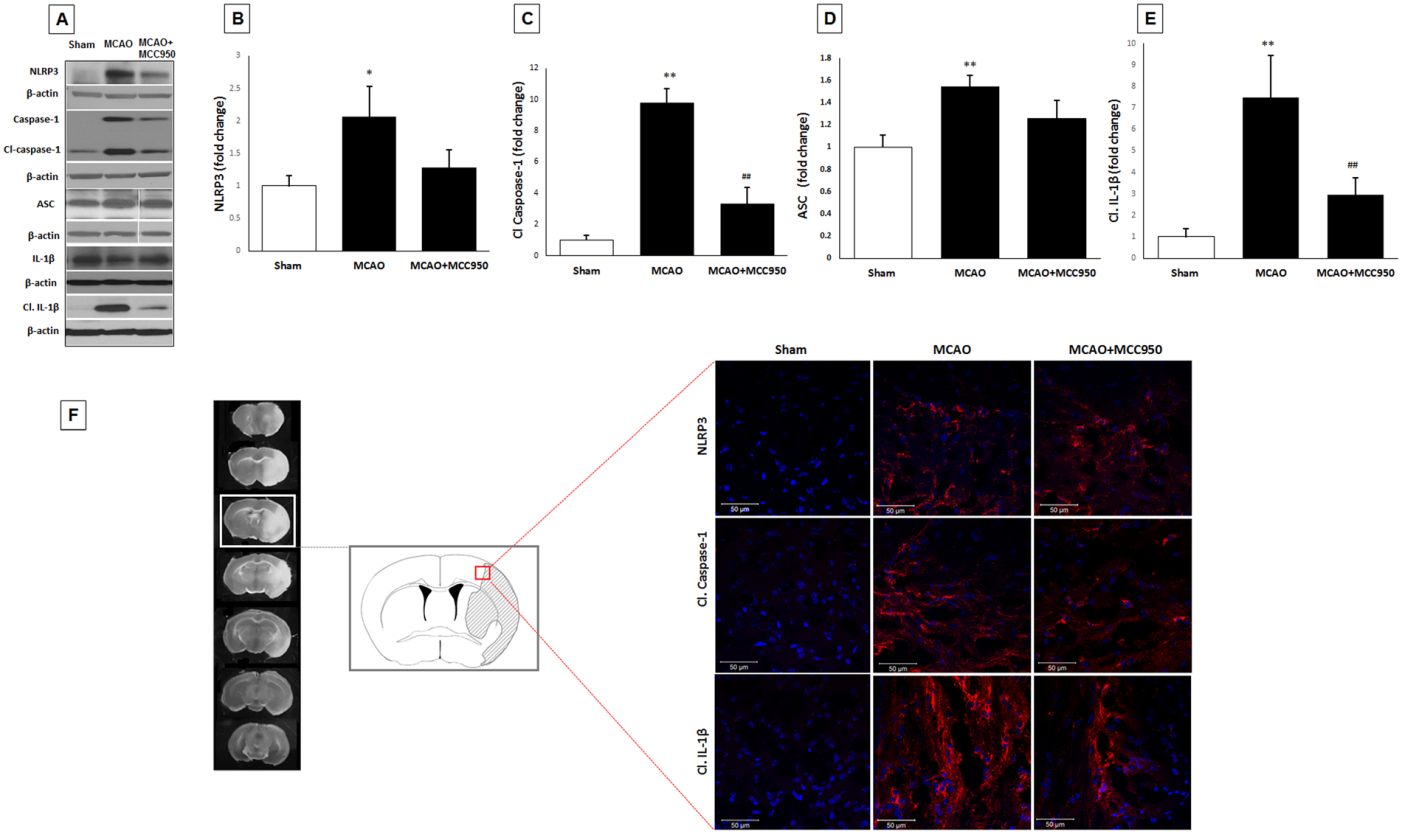
MCC950 treatment inhibits NLRP3 inflammasome components (cleaved caspase-1 and cleaved IL-1β) at 24 h post-MCAO. Representative (**A**) and quantitative analysis of NLRP3 (**B**), cleaved caspase-1 (**C**), ASC (D) and cleaved IL-1β (**E**) levels demonstrate the corresponding differences in penumbral region at 24 h after MCAO. MCC950 treatment reduced cleavage of caspase-1 and IL-1β maturation as consequents of NLRP3 inhibition. The changes were then confirmed in frozen section samples obtained from peri-infarction areas (penumbra) (**F**) immunostained with NLRP3, cleaved caspase 1 and mature IL-1β. Blue staining: CNS cells nuclei; Red staining: probing of the corresponding primary antibodies. Values are expressed as mean ± SEM (n = 5). *p < 0.05, **p < 0.01 vs sham, ^##^p < 0.01 vs saline treated MCAO animals.

The principal constituents of NLRP3 inflammasome assembly as well as the consequent caspase-1 and IL-1β production were analyzed to determine the inflammasome activation in penumbral regions. NLRP3 inflammasome activation following stroke is established in earlier studies to contribute to stroke injury through the pro-inflammatory cytokines (e.g. IL-1, IL-18) as well as the pleiotropic effects of cleaved caspase-1 in mediating pyroptosis and apoptosis^29^. The expression of NLRP3 inflammasome products cleaved caspase-1 and cleaved IL-1β significantly (*p* < 0.05) elevated at 24 h after MCAO compared to shams (Fig. 2A–E). However, there was no difference in the expression of IL-1β and IL-18 in response to MCAO (Supplementary information Fig. 1). These findings were further confirmed with elevated immunopositive signals of NLRP3, caspase-1 and IL-1β in peri-infarct area of brain sections in saline-MCAO group only (Fig. 2F). MCC950 treatment prevented the activation of NLRP3 as determined by reduced cleavage of caspase-1 and the subsequent release of IL-1β after MCAO (*p* < 0.01), however it did not provide a discernible effect on NLRP3 or ASC protein levels.

### MCC950 decreases phospho-NFκBp65/NFκBp65 ratio as well as TNF-α expression after MCAO

**Figure 3:**
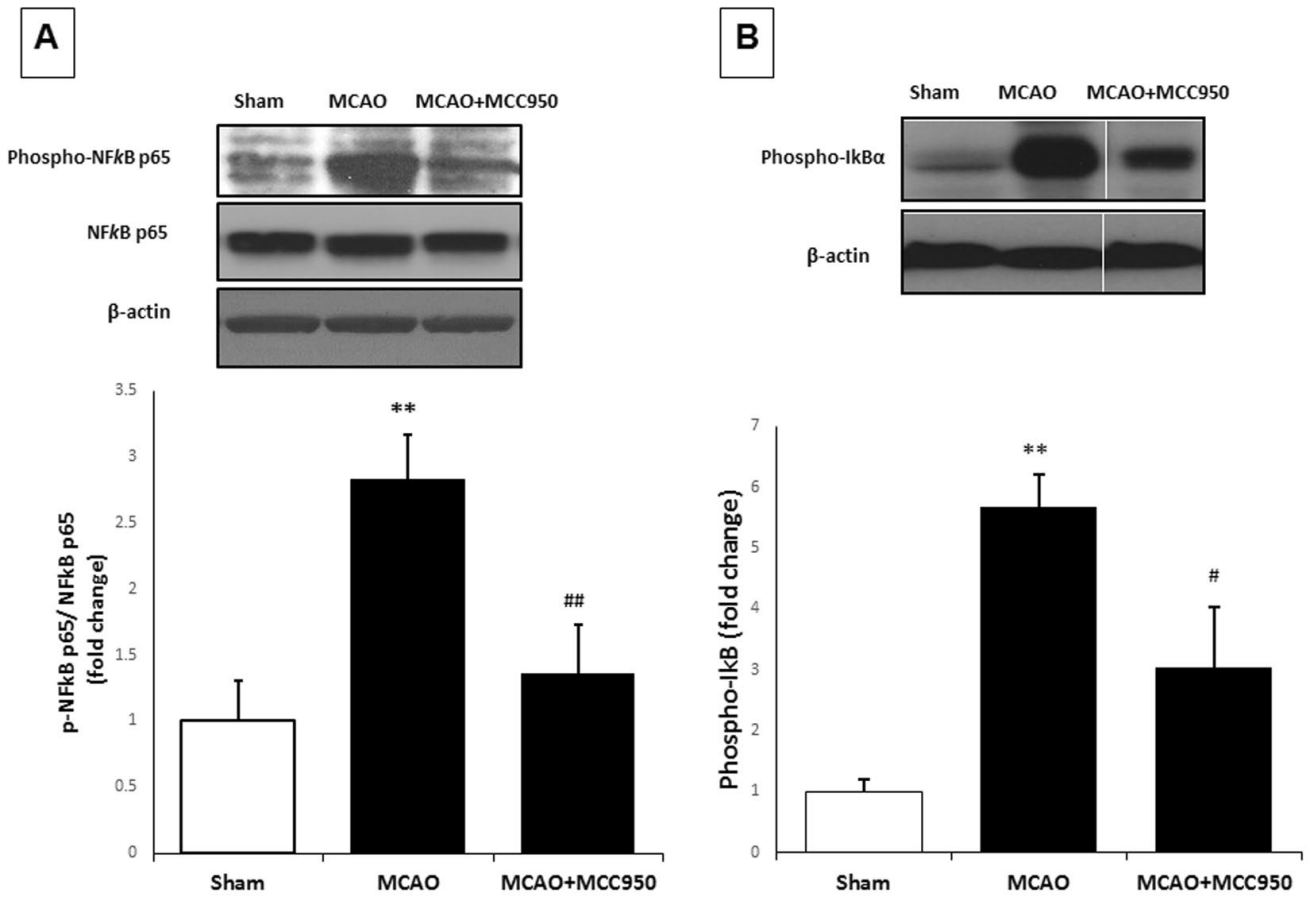
MCC950-treatment attenuates the activation of NFκBp65 at 24 h post-MCAO. Following 24 h I/R, mice brains were subjected to immunoblotting for (**A**) NFκBp65, phospho-NFκBp65 (p-NFkBp65) and (**B**) Phospho-IκBα in penumbral region to obtain phospho-NFκBp65/ NFκBp65 ratio as a general index for NFkBp65 transcriptional activity. Increased expression of Phospho-IκBα confirm the activation of NFkB transcriptional activity. MCC950 treatment was associated with significant fall in phospho-NFκBp65/ NFκBp65 ratio and Phospho-IκBα. Values are expressed as mean ± SEM (n = 5). **p < 0.01 vs sham, ^#^p < 0.05, ^##^p < 0.01 vs saline treated MCAO animals.

We further elucidated the effect of MCC950 on phospho- NFκBp65/NFκBp65as an index for activation of NFκB, the transcriptional regulator of inflammatory genes at 24 h after MCAO (Fig. 3A). Our immunoblotting showed phospho- NFκBp65/NFκBp65is significantly (P < 0.05) elevated in MCAO group compared to shams, which was further inhibited with MCC950 treatment Increased activation of NFκB is further confirmed by enhanced expression phosphor-IκBα, an endogenous negative regulator NFκBp65. Expression of Phospho-IκBα was significantly elevated (p < 0.01) at 24 h after MCAO and inhibited by treatment with MCC950 (Fig. 3B). Increased phosphorylation of IκBα causes their proteasomal degradation thereby induces increased transcriptional activity of NF-κB^30^.

**Figure 4:**
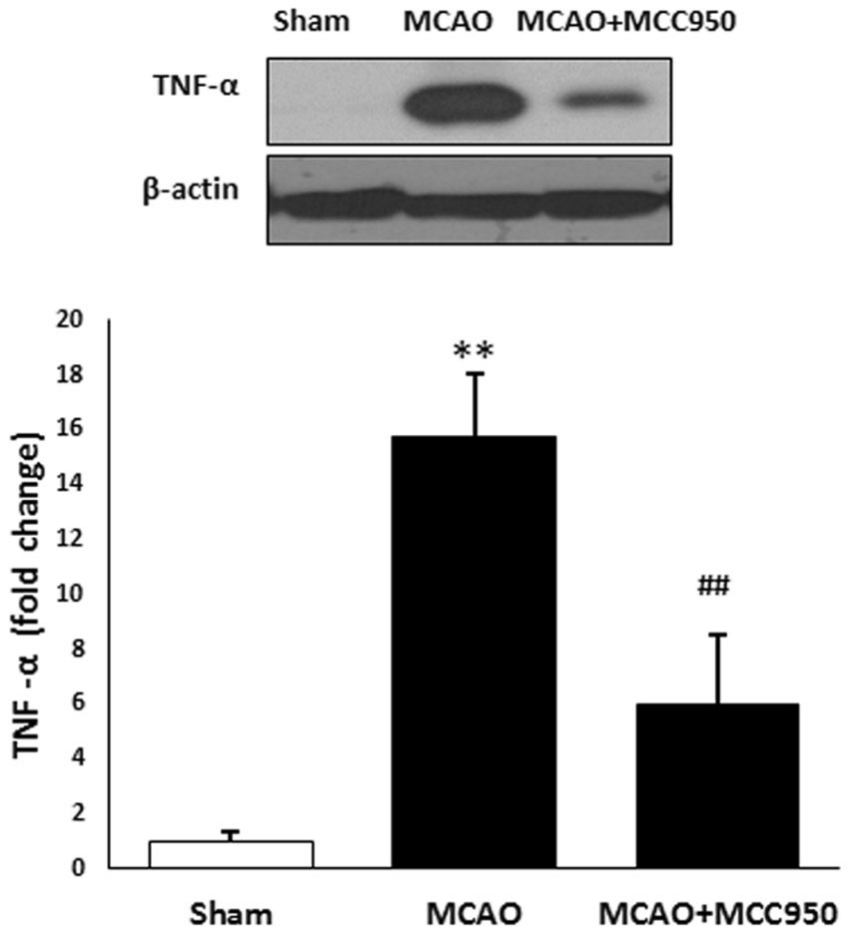
MCC950-treatment attenuates the activation of TNF-α at 24 h post-MCAO. Following 24 h I/R, mice brains were subjected to immunoblotting of TNF-α in penumbral region as a general index for stroke-associated inflammation. MCC950 treatment was associated with remarkable fall in the TNF-α expression. Values are expressed as mean ± SEM (n = 3–5). **p < 0.01 vs sham, ^##^p < 0.05 saline vs treated MCAO animals.

The effect of MCC950 on TNF-α, a pleiotropic cytokine that rapidly upregulates in the brain after injury was also examined. Our immunoblotting showed that TNF-α is highly expressed in MCAO group more than ten folds as much as that in shams (Fig. 4). MCC950 treatment remarkably reduced TNF-α in stroke animals from 15.74 ± 2.33 to 6.01 ± 2.55 (arbitrary unit) with p value of 0.01.

### MCC950 attenuates activation of caspase-3 and PARP after MCAO

**Figure 5:**
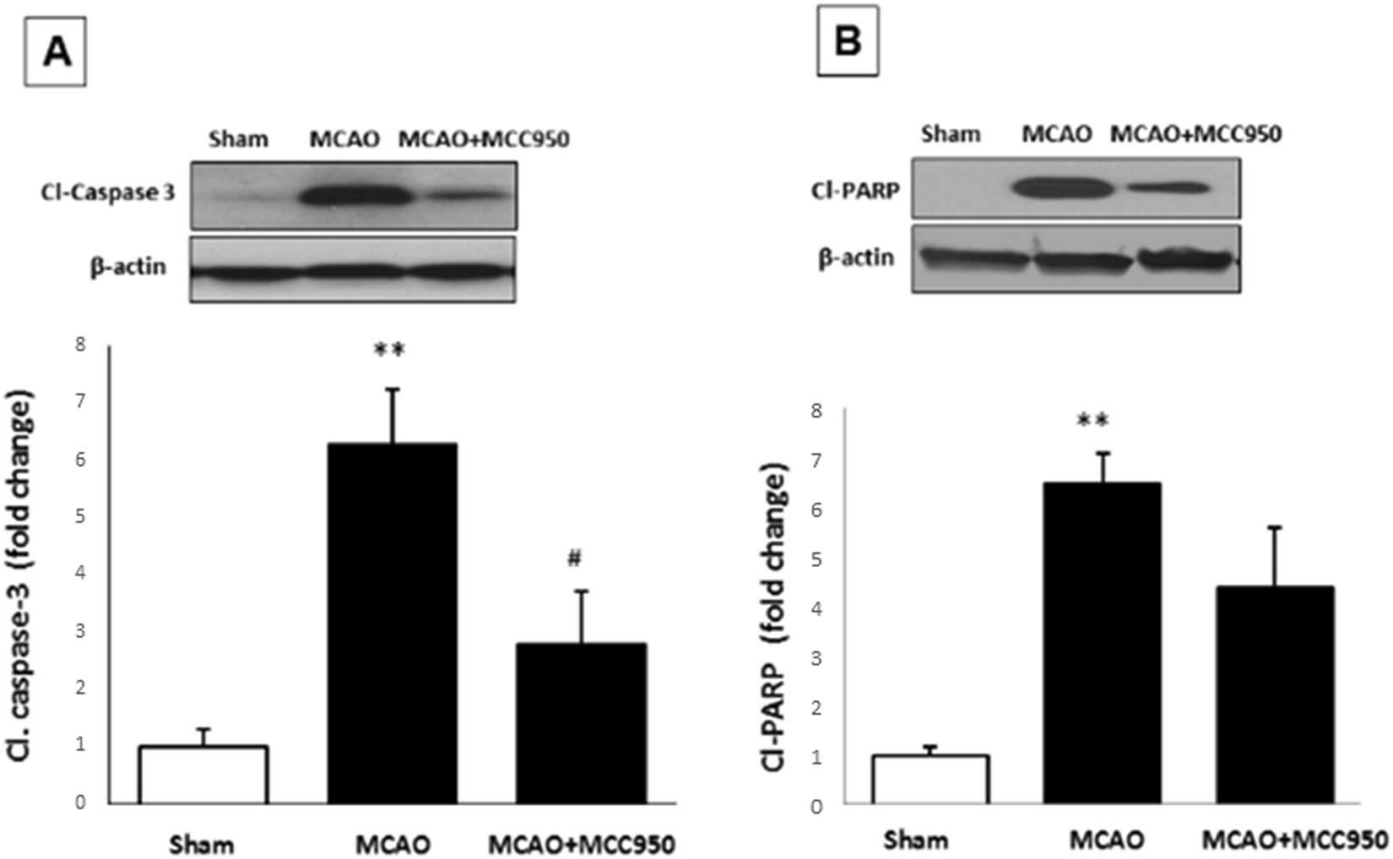
MCC950 treatment partially reduces the expression of cleaved caspse-3 and PARP at 24 h post-MCAO. Following 24 h I/R, penumbral cerebral cortex in mice brains were immunoblotted for assessment of pro-apoptotic markers. According to the representative immunoblots, expression of cleaved caspase-3 (**A**) and cleaved PARP (**B**) were significantly increased, and was further partially inhibited with the treatment MCC950. Values are expressed as mean ± SEM (n = 5). **p < 0.01 vs sham. ^#^p < 0.05 saline vs treated MCAO animals.

To examine the effect of MCC950 on neural apoptotic pathways, we next estimated activation of the pro-apoptotic PARP and caspase-3 at 24 h after MCAO (Fig. 5A,B). In the ischemic condition, the activation of PARP following DNA damage might contribute to caspase-3 activation. The expression of cleaved PARP and cleaved caspase-3 were significantly (P < 0.01) increased after MCAO compared to shams. Treatment with MCC950 significantly reduced activation of caspase-3 (p < 0.05) expression, parallel with marginal decrease in activation of PARP (Fig. 5).

## Discussion

NLRP3 inflammasomes are known as key intracellular regulators of innate responses to inflammation and infection^31^. Our recent study indicated that genetic or pharmacological ablation of the thioredoxin interacting protein (TXNIP) is associated with suppression of NLRP3 inflammasome and attenuation of stroke outcome^21^. The present data shows that specific NLRP3 inflammasome inhibition by MCC950 alleviates infarct size, edema, hemorrhage and behavioral deficits in suture MCAO model of stroke. Consistent with earlier report demonstrating reduced NLRP3 inflammasome activation coincident with neuroprotective modulations^19,29,32^, these findings support our presumption that inhibition of NLRP3 inflammasome is the key effector of TXNIP ablation as well as stroke induced injury.

Increasing evidence implicates the NLRP3 inflammasome and its activation/release products; ASC specks, caspase-1 and IL-1β in the pathogenesis of neurodegenerative disease and stroke^33–35^. The comprehensive study by Fann and colleagues principally showed that the levels of NLRP3-inflammasome and IL-1β were increased in cellular and animal models of stroke^29^. The implication was further supported by Yang et. al data demonstrating NLRP3 deficiency ameliorated ischemic injury in cellular and animal models of stroke [5]. In an attempt to define relevant pharmacological tools, Fann’s research team later introduced intravenous immunoglobulin as a protective approach against experimental stroke by a mechanism involving suppression of NLRP3-inflammasome activity^29^. In our earlier report, we also observed similar mitigating effects for resveratrol as an in indirect inhibitor of NLRP3 inflammasome^36^. Importantly, following development of MCC950 in 2015 as a selective potent MCC950 inhibitor with exceptional bioavailability and kinetic attributes, many studies have been focused to unravel the due therapeutic applications. The very recent findings of Ye *et al*.^20^ has already demonstrated notable therapeutic effects on *in vitro* and photothrombotic *in vivo* models of stroke. Dosed with daily MCC950 (50 mg/kg, i.p.), the animals showed significantly reduced cerebral infarction and neurological deficits day 3 post-stroke. Such alleviations were all in parallel with reduced NLRP3/ASC/Caspase-1 levels within the ischemic core, with its defined less genes expression potentials.

However, we have the same conclusion supporting MCC950 benefits in experimental stroke, there are a few noteworthy differences. Confirmed by statistics, our animals subjected to MCAO and MCC950 treatment in the acute phase (50 mg/kg, i.p., 1 and 3 h post-stroke), came up with remarkable protection in infarction (p < 0.01 vs p < 0.05) and fast-healing neurological function. This might be simply ascribed to our early-started treatment aside our particular animal model. Indeed, early expression of IL-1β in areas of focal neuronal injury underlines it as the major form of IL-1 contributing to inflammation early after cerebral ischemia^37^. Indicating the role of NLRP3 inflammasome to cytokine release in the acute phase, this may explain how treatment with MCC950 producing rather remarkable effect in our investigations.

Furthermore, there is a strong emphasis on the proof of concept studies in different animal models, according to STAIR protocols. The photothrombotic stroke model used in Ye et. al. study has several advantages including high survival rate though, it suffers from small infarct sizes limited to cortices, and above that, lack of defined penumbral region both of which may hurdle appropriate translational conclusion^38^. Specifically, while dealing with inflammasome and inflammation concepts, penumbra is the main region to focus. Therefore, with our particular MCAO model and penumbral brain sampling, we could track a remarkable caspase-1/IL-1β repression followed by slightly abolished PARP and caspase-3 cleavage, addressing confined inflammation and degeneration in MCC950 treated brains. Furthermore, we detected a 10-fold increase in penumbral TNF-α in MCAO stroke brains associated with a dramatic NFκBp65 activation. This might be suggestive of TNF-α induced NLRP3 inflammasome priming though, TNF- α still have other ways to induce oligomerization of the pre-existing inflammasome constituents e.g. trough mediating oxidative stress^39,40^. According to our findings, in contrary to Ye obtained data; we did not detect any change in NLRP3 and ASC levels. Basically, MCC950 is defined to specifically block the assembly of NLRP3 constituents. Therefore, one may not expect transcriptional changes in proteins building NLRP3 inflammasome. Nevertheless, as we detected less pro-caspase 1 levels in MCC950 stroke animals, the eventual modulation of inflammatory cytokines arguably may alter transcripts of pro-IL-1/caspase 1 namely through NFκB modulation.

Evidence of NLRP3 implication in cerebrovascular diseases is not confined to preclinical data. Conspicuously, recent randomized trials imply people with some specific NLRP3 genotype has been shows more prone to stroke and transient ischemic attacks^41,42^. This might underline TXNIP/NLRP3 inflammasome central function in endothelial integrity to drive atherosclerotic changes^43^. Although this may somehow address the reduced hemorrhagic transformation in our MCC950 stroke animals, it is hard to differentiate whether it is the direct effect of MCC950 or the confined inflammatory responses.

Nevertheless, there are some restrictions on our interpretations yet to be addressed in further works. The limitations of this study are mainly those associated with the use of a single endpoint at 24 h post-stroke and intraluminal suture model of MCAO. Further pertinent studies are essential to better realize the effect of MCC950 in a combinatorial medical approach including recanalization by tissue plasminogen activator (tPA) in embolic stroke models to analyze cerebrovascular damage.

**Figure 6:**
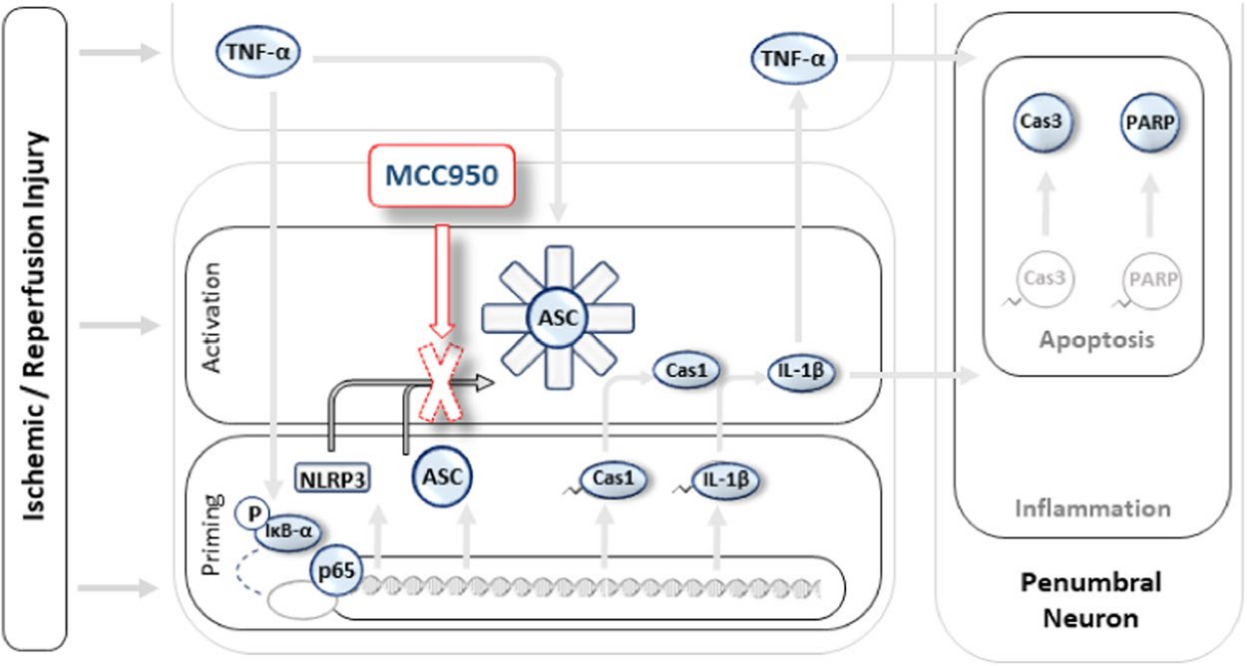
Pictorial description of MCC950 therapeutic effects on experimental stroke. NLRP3 inflammasome activation is demonstrated in background pale illustrates with the colored objects representing the evaluated index molecules to address MCC950 effect. In a simplified look, stroke triggers several deteriorating signals leading to NLRP3 inflammasome priming or activation. The priming step which largely depends on transcriptional activity of NFκB, works to build up all the prerequisite NLRP3 components (NLRP3, ASC, pro-caspase-1 and pro-IL-1β) along other pro-inflammatory molecules like TNF-α. The activation process instantly follows the priming step to assemble the NLRP3 inflammasome and subsequent caspase-1 cleavage and IL-1β maturation. IL-1β may instigate a wide range of inflammatory responses which parallels IκBα phosphorylation, phospho-p65 transcriptional activity and TNF-α over production. Apoptotic cell death involving classic caspases and PARP follow the inflammatory propagation. MCC950 treatment produces therapeutic benefits in experimental stroke. This is attributed to specific inhibition of NLRP3 oligomerization leading to reduced caspase-1 and IL-1β activation and protection against pro-apoptotic and pro-inflammatory cascades and ischemic insult escalation. Abbreviations: NLRP3: NOD-like receptor protein-3; IL-1β: Interleukin 1 beta; TNF-α: Tumor necrosis factor alpha; p65: NFκBp65 subunit, IκBα: inhibitor of kappa B-α

The present study concludes that MCC950 shows appropriate *in vivo* efficacy to repress NLRP3/Caspase-1/IL-1 signaling enough to improve acute stroke outcomes in a MCAO model of stroke (Fig. 6). Given that few approaches are available in managing stroke suspects before admission, investigating efficient oral therapeutics like MCC950 might be promising in conscious stroke suspects. Providing supportive evidence for NLRP3 involvement in acute injury following stroke, these findings further suggest that NLRP3 inflammasome may represent a promising imminent target to develop newer therapeutics.

## Experimental Procedures

### Animals and experimental groups

Wild-type C57Bl/6 mice (Jackson Laboratory, Bar Harbor, ME, USA) were used in the study. All experiments were conducted according to procedures approved by the Institutional Animal Care and Use Committee (IACUC) at UTHSC, Memphis TN. The study are reported here in accordance with the ARRIVE (Animal Research: Reporting *in Vivo* Experiments) guidelines^44^. The animals were housed in standard humidity (45–50%) and temperature (21–25 °C) and 12-h light/dark cycle with food and water ad libitum. Adult male (8–10 weeks) mice were quarantined for at least 7 days before the experiment when they were subjected to MCAO or sham surgery. Wild-type C57Bl/6 mice were assigned to three different experimental groups including sham and MCAO animals with saline or MCC950 treatment. MCC950 (Sigma, USA) was dissolved in sterile saline and administered (50 mg/kg, i.p.) 1 h and 3 h post-occlusion. The MCC950 dosage was determined based on previous published studies^22,45^.

### Induction of focal cerebral ischemia

Adult animals (22–25 gm) were subjected to middle cerebral artery occlusion (MCAO) using the intraluminal suture model as described previously^46^. Briefly, animals were anesthetized using 2–5% isoflurane inhalation. Middle cerebral artery occlusion (MCAO) was achieved using a 6–0 silicon-coated nylon suture (Doccol, Sharon, MA), advanced into the internal carotid artery to block the origin of the middle cerebral artery. After 1 h, animals were re-anaesthetized to remove the sutures and allow reperfusion in ischemic brain areas. Animals were kept in a 37 °C for their comfort and recovery after surgery.

### Assessment of functional Neurological deficit score

Neurological deficits were evaluated in a blinded manner after 24 h I/R just before animals were euthanized for *ex-vivo* evaluations. Using the modified neurological deficit score^21^, animals with no apparent deficits obtained 0; signs of forelimb flexion, 1; reduced resistance to push, 2 and with circling, 3. For consistent MCAO completion, only animals with a score of ~3 at reperfusion were included in further analysis.

### Assessment of infarct size and edema

Infarct size, edema and hemoglobin content (hemorrhage) were measured by a blinded investigator. After 24 h of MCAO, animals were deeply anaesthetized with ketamine/ xylazine mixture (85% and 15%, respectively) and transcardially perfused with ice cold PBS. Animals were then decapitated and their brains collected. Seven 1-mm thick coronal sections from each brain were stained with 2% TTC solution (2,3,5-triphenyltetrazolium chloride- Sigma-Aldrich, St. Louis, MO) for 2 min and scanned. Infarction and total hemispheric areas were blindly measured using Image J software and corrected for edema based on recent optimizations using the formula: [(infarct V/ipsilateral V) × contralateral V]^47^. Hemispheric edema was calculated as the area difference in the ischemic hemisphere compared to the contralateral hemisphere.

### Evaluation of hemoglobin content

Cerebrovascular disruptor and the subsequent blood cells infiltration to the brain tissue was quantified using a colorimetric hemoglobin detection assay (QuantiChrom Hemoglobin Assay Kit, BioAssay Systems; Haywood, CA) to address hemorrhagic transformation after stroke. Ipsilateral TTC-stained brain samples were separated and homogenized in a 10% glycerol-Tris buffer saline solution containing 0.5% Tween-20. Samples were subjected to the kit reactions yielding a uniformly colored hemoglobin and read at 562 nm using a standard microplate reader (Synergy HT, BioTek instruments). Hemoglobin concentration was calculated according to the manufacturer’s instructions and recorded in µg/dL based on the standard samples.

### Western blotting

For WB analysis, we used peri-infarct (penumbra) cortical regions. Using a brain matrix, the brains were rapidly dissected into 4.0 mm coronal sections (approximately 0.5 mm and −3.5 mm from bregma). Brain tissue was homogenized and processed for western blotting as previously described. Fifty-microgram of proteins were loaded in each lane and separated followed by transfer to nitrocellulose membranes. The membranes were blocked for non-specific binding and probed with primary antibodies against NLRP3, Caspase-1, ASC (1:1000; AG-20B-0014; AG-20B-0042; AG-25B-0006 Adipogen life sciences), cleaved IL-1β, Caspase-3, Phospho- NFκBp65, NFκBp65 (1:1000; CST-12242; 9664; 3033; 8242; Cell signaling technology), Cleaved PARP, Anti IL-18, IL-1β (1:1000; ab32064; ab71495; ab9722; Abcam, USA), phospho IκB (1:1000; Santa cruz biotechnology, SC8404) at 4 °C for overnight. Following TBS-T washes, the membranes were incubated with horseradish peroxidase–conjugated secondary antibody (1: 10,000; Sigma). The bands were then visualized by means of an enhanced chemiluminescent substrate system (Thermo fisher scientific). Protein levels were analyzed densitometrically, using Image J software and were normalized to loading controls, and expressed as fold change.

### Immunofluorescence Staining

At 24 h after tMCAO, mice were anesthetized with ketamine/xylazine and transcardially perfused with 30 mL of PBS followed by 50 mL of 10% formalin (Fischer Scientific). Brains were removed and post-fixed in the same fixative overnight at 4 °C and then with 30% sucrose in PBS for 72 h. The brains were sectioned in the coronal plane at a thickness of 10 μm. Sections were blocked with Serum-Free Protein Block (X0909, DAKO) followed by incubation with primary antibodies against NLRP3, cleaved caspase-1 (1:200; AG-20B-0014, 1: 250; AG-20B-0042, Adipogen life sciences) and cleaved IL-1β (1:100; CST-12242, Cell signaling technology, USA) overnight at 4 °C in a humid chamber. After washing, slides were incubated with fluorescent anti mouse secondary antibodies (1:200; 072–04–18–03; Dylight-549, KPL) for 1 h at room temperature and mounted with ProLong™ Diamond Antifade Mountant with DAPI (Invirogen), and viewed using a Zeiss 710 confocal laser scanning microscope. Negative controls were prepared by omitting the primary antibodies.

### Statistical Analysis

The results were expressed as mean ± SEM. Differences among experimental groups were evaluated by student’s t test or ANOVA followed by Tukey’s post-hoc test. Significance was defined by a p < 0.05.

### Data Availability

The datasets generated during the current study are available from the corresponding author on a reasonable request.

## Electronic supplementary material

Supplementary information
