# Supplementary material for: Inhibition of the NLRP3-inflammasome as a potential approach for neuroprotection after stroke
Source: Sci Rep. 2018 Apr 13;8:5971. doi: 10.1038/s41598-018-24350-x (PMC5899150; doi:10.1038/s41598-018-24350-x)
Supplement: Supplementary file 1 — Supplementary information [file 41598_2018_24350_MOESM1_ESM.pdf]

Inhibition of the NLRP3-inflammasome as a potential approach for neuroprotection after stroke

Saifudeen Ismael,[1] Liang Zhao,[1] Sanaz Nasoohi,[1,2] Tauheed Ishrat[1,3]*

Effect of MCC950 on Cleaved IL-18 and IL-1β at 24 h after MCAO

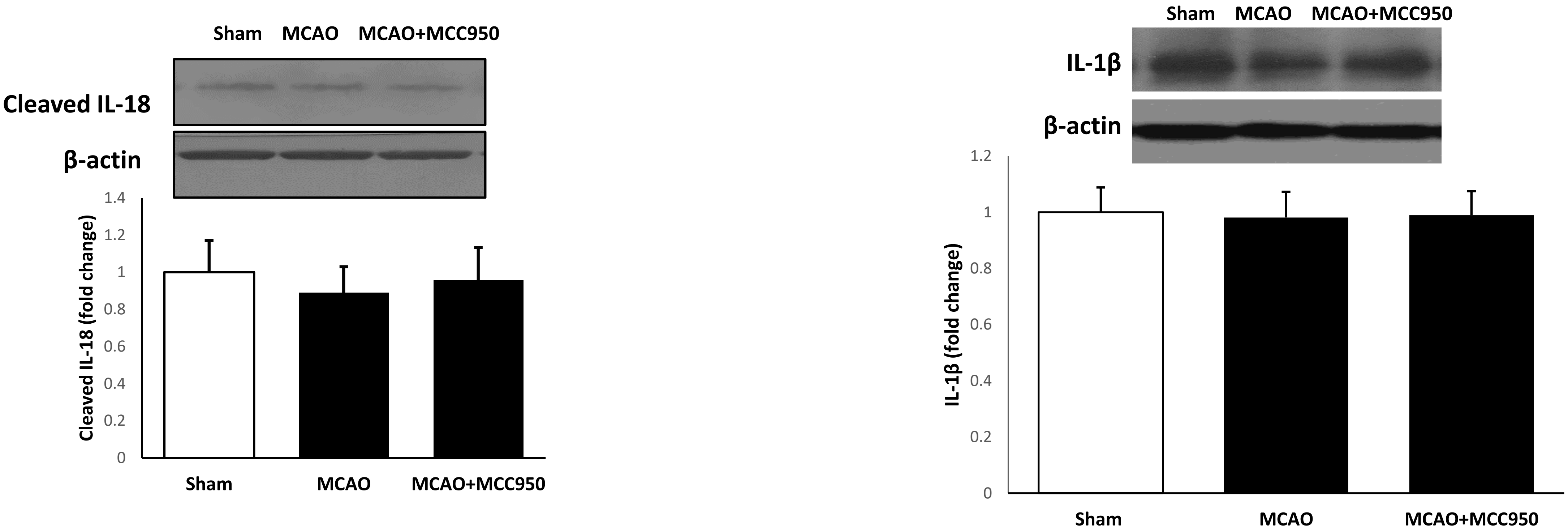

Figure 1: Expression of Cleaved IL-18 and **IL-1β following treatment with MCC950, 24 h post-MCAO.** There was no difference in the expression of IL-1β and IL-18 in response to MCAO and MCC950 treatment

Inhibition of the NLRP3-inflammasome as a potential approach for neuroprotection after stroke

Saifudeen Ismael,[1] Liang Zhao,[1] Sanaz Nasoohi,[1,2] Tauheed Ishrat[1,3]*

All Full-length Blots

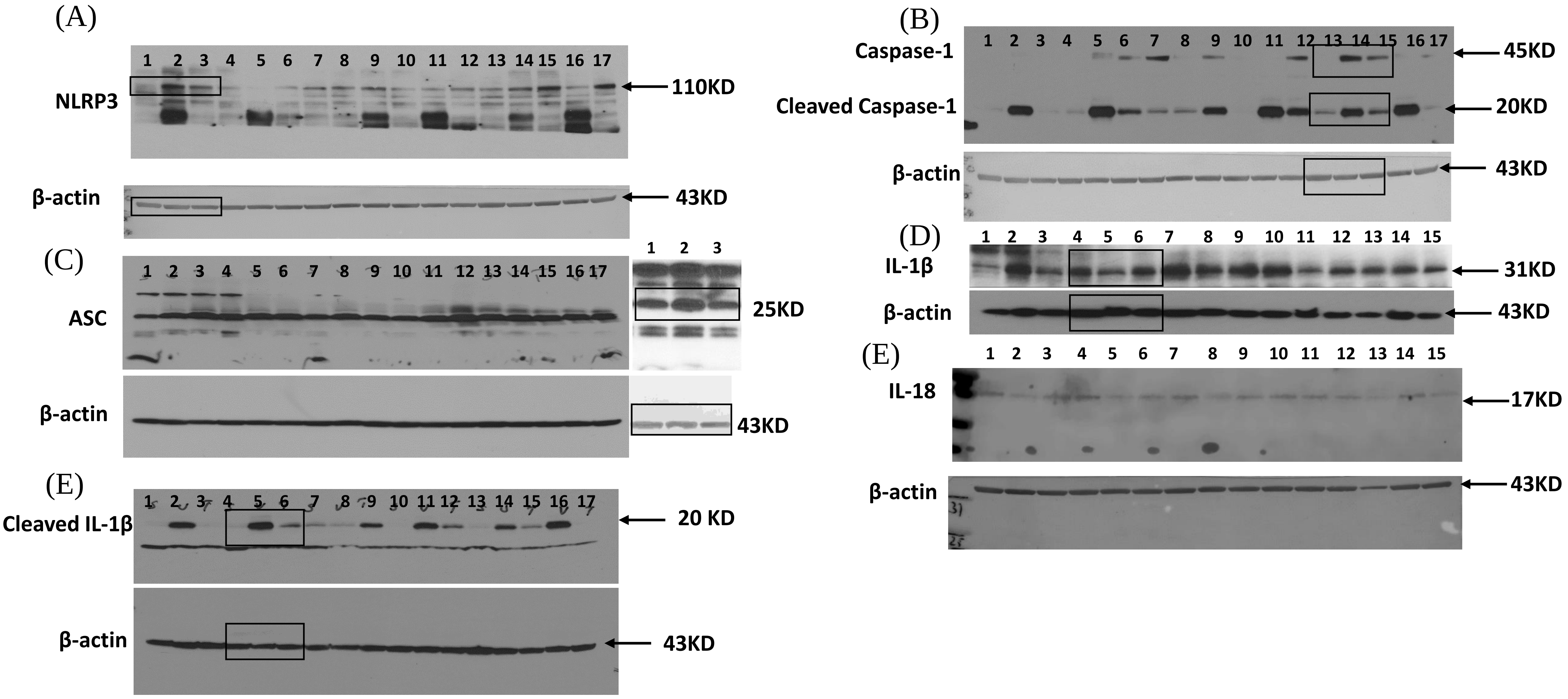

Figure 2: Expression of NLRP3 inflammasome components(A) NLRP3 (B), Cleaved caspase-1 (C), ASC (D) and IL-1β, following treatment with MCC950, 24 h post-MCAO.

Sham: 1, 4, 7, 10, 13; **TMCAO:** 2, 5, 8, 11, 14, 16; **TMCAO+MCC950:** 3, 6, 9, 12, 15, 17

The representative images used in manuscript are highlighted with box.

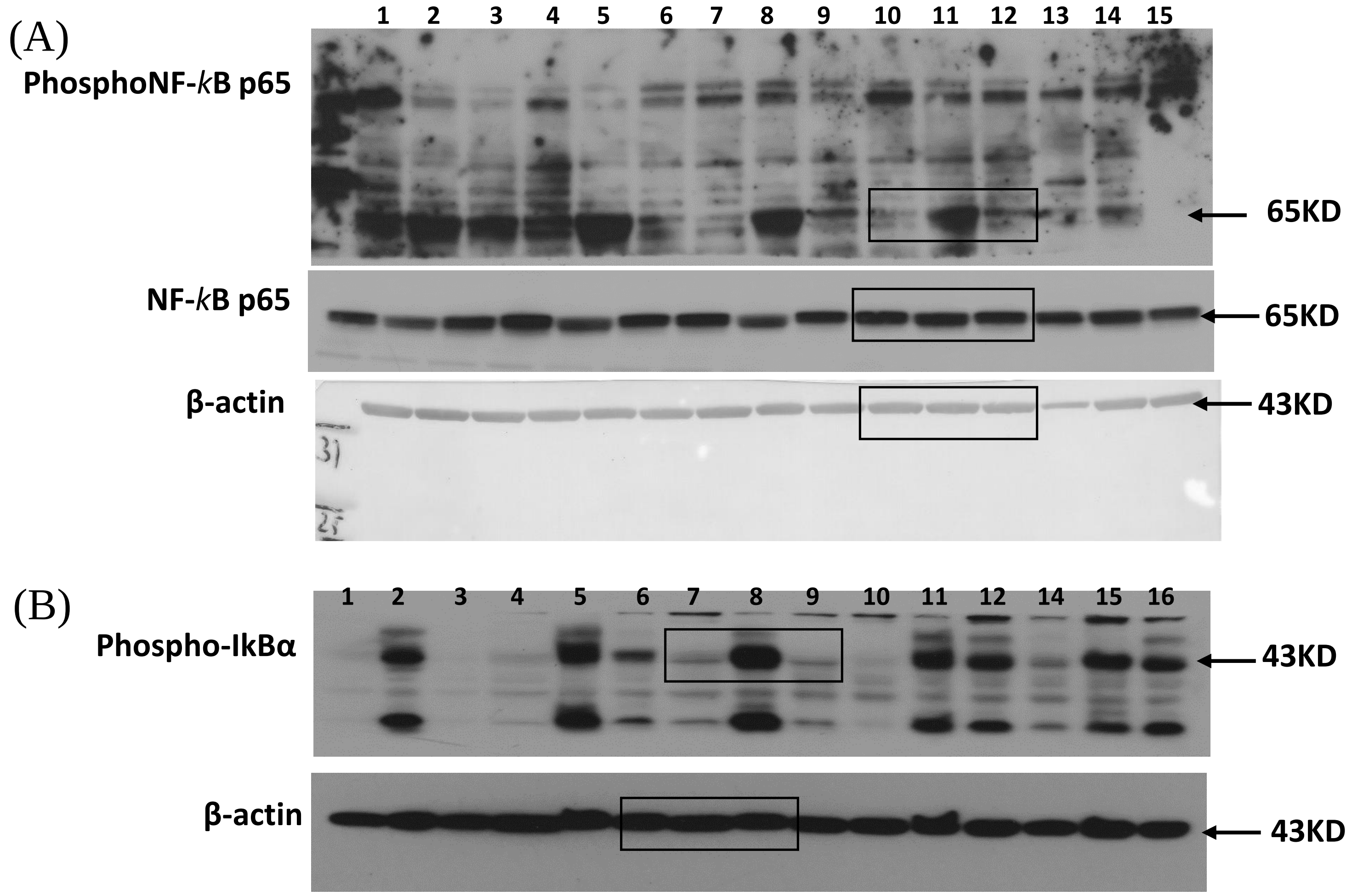

Figure 3: Expression of phospho-NFkBp65 and NFBp65 (A) phospho-IkBα (B) following treatment with MCC950, 24 h post-MCAO. **Sham:** 1, 4, 7, 10, 13; **TMCAO:** 2, 5, 8, 11, 14, 16; **TMCAO +MCC950:** 3, 6, 9, 12, 15,

The representative images used in manuscript are highlighted with box.

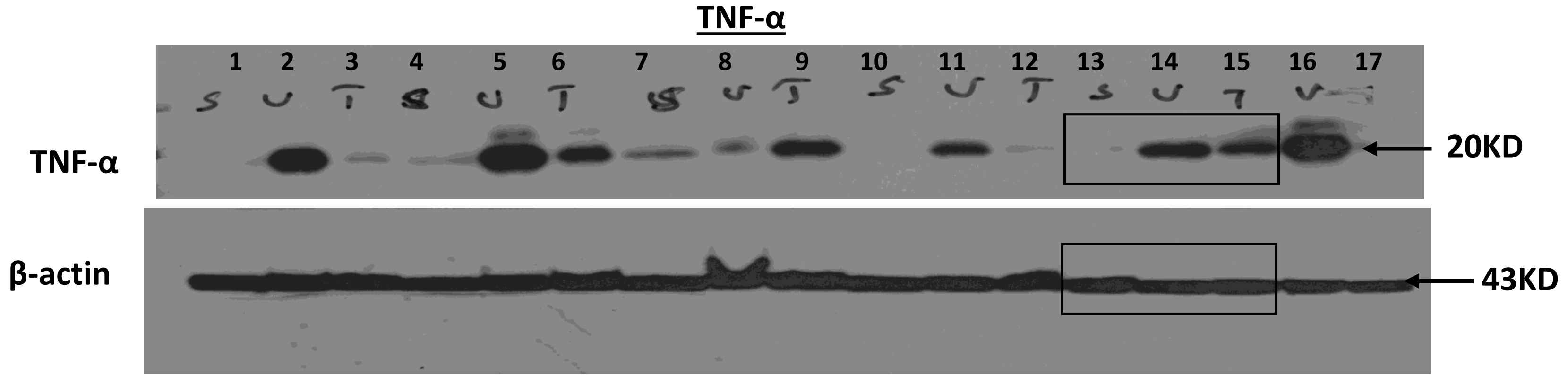

Figure 4: **Expression of TNFα, following treatment with MCC950, 24 h post-MCAO.**
Sham: 1, 4, 7, 10, 13; **TMCAO:** 2, 5, 8, 11, 14, 16; **TMCAO+MCC950:** 3, 6, 9, 12, 15, 17
The representative images used in manuscript are highlighted with box.

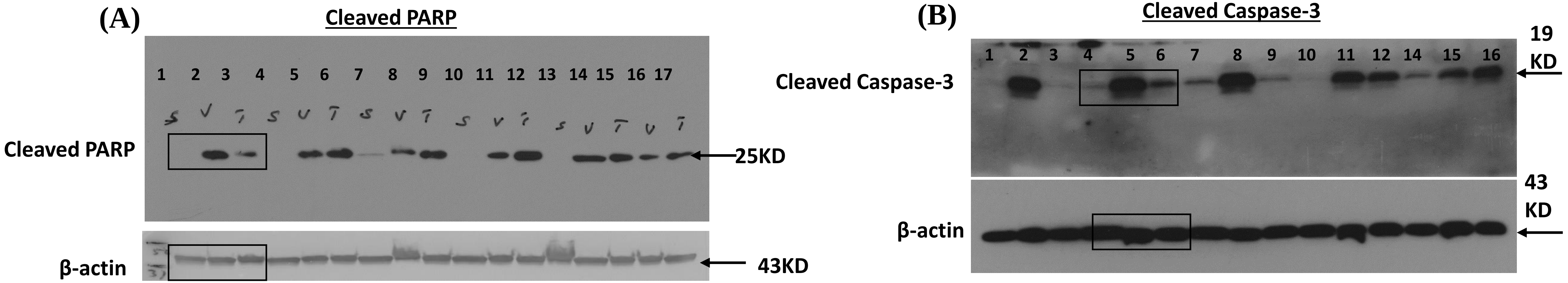

Figure 5: **Expression of (A) Cleaved PARP and (B) Cleaved caspase 3, following treatment with MCC950, 24 h post-MCAO.**
Sham: 1, 4, 7, 10, 13; **TMCAO:** 2, 5, 8, 11, 14, 16; **TMCAO+MCC950:** 3, 6, 9, 12, 15, 17
The representative images used in manuscript are highlighted with box.
